# Mapping of biodiversity hubs and key ecosystem services as a tool for shaping optimal areas for conservation

**DOI:** 10.1371/journal.pone.0253151

**Published:** 2021-08-17

**Authors:** Olivier Clement Gatwaza, Xiangrong Wang

**Affiliations:** School of Landscape Architecture, Beijing Forestry University, Beijing, P.R.China; Qinghai University, CHINA

## Abstract

Most National Parks (NP) and nature reserves in Rwanda have been established opportunistically in the early 1900s, without clear consideration of ensuring the protection to all threatened different taxonomical or functional groups, such as vegetation, invertebrates, fish, and birds. With the increasing conservation objectives, raised expectations into Protected Areas (PA), and within a more challenging environmental context, it is important to identify biodiversity hubs and key areas for Ecosystem Services (ES) to maximize the efficiency of conservation efforts by assisting priority areas under threats. To date, no comprehensive analysis, to the best of our knowledge has been done to assess both biodiversity and ES in Rwanda. This is a notable gap, considering that global-scale research suggests that the spatial overlap between biodiversity targets and ES is low. This study reports a nationwide assessment, mapping the richness of threatened species and three key ES Carbon Storage, Water Quantity, and Water Quality. Our analysis has shown that PAs are neither perfectly delineated to protect biodiversity nor key ES. The state of PAs offers a taxonomic protection bias in favor of mammals and birds but leaves many endangered species in other taxonomic groups in collapsing and unprotected small ecosystems scattered around the country. Rwanda’s PAs cover important carbon stock but can do better at securing higher water balance regions and clean water sources. We propose an improvement of the NP system in Rwanda to help guide the economic development along a path of green growth and ensures the well-being of both people and nature. Locating biodiversity hubs and key ES can help to connect conservationists, local people, and governments in order to better guide conservation actions.

## 1 Introduction

The degradation of natural ecosystems relentlessly continues to threaten the long-term survival of many species around the world [1]. There is general consensus that establishing Protected Areas (PA) is the major strategy for conserving biodiversity pools and the provision of ES worldwide [1, 2]. PA are now created not only to conserve iconic landscapes, seascapes and to provide habitat for endangered wildlife but also to contribute to many other functions among which are to sustain the livelihood of local communities, to support national economies through tourism revenues, to replenish fisheries, and to play a key part in the mitigation of climate change. Although the expanded role of PA may have fueled their establishment, their constantly widening focus makes them vulnerable to accusations of failure to achieve one or more of these objectives [3]. Many PA are either too small to serve their intended functions, too damaged to support conservation purposes, or are not located in areas of the most significant conservation value. Serious deficiencies in the structure and location of PA were raised by numerous studies in different parts of the world. Liang et al. [4] have pointed out loopholes at PA location and top priority conservation areas, while Xu et al. [5] found a surprising divergence between the location of established PA and endangered biodiversity hubs on one hand and key ES on the other hand. Myers [6] coined the term ‘biodiversity hotspot’ as a biogeographic region characterized both by exceptional concentrations of species with high levels of endemism and by serious levels of habitat loss. According to Myers et al. [7], and Joppa et al. [8] to qualify as a hotspot, a region must meet two strict criteria: It must contain at least 1,500 species of vascular plants (> 0.5% of the world’s total) as endemics, and it has to have lost at least 70% of its original habitat. Biodiversity hotspots, as well as other global biodiversity priority schemes such as Global ecoregions, and High-Biodiversity Wilderness Areas (HBWAs) primarily aim to ensure the efficiency use of conservation investments [9, 10]. For decades, conservationists have recognized that rare plants are clustered into small and isolated habitat patches produced by landscape features [11]. As Myers et al. [7] put it, Rwanda is among regions that appear to feature exceptional plant endemism and exceptional threat, but that are not sufficiently documented to meet the hotspots criteria. For this reason, ‘biodiversity hub’ terminology will be used to express a biogeographic region with significant levels of endemic biodiversity that is threatened by anthropologic activities Weaknesses at protecting such important regions have been raised by different authors. Mountain ranges, for instance, constitute biodiversity hubs, particularly for montane species but existing protection systems fail to fully capture montane biodiversity patterns or facilitate species range shifts [12–14]. By contrast, the common preference to protect ‘wild’ areas, thanks to their easier opportunities for the future expansion of PA compared to the greater challenges of expansion into human-dominated landscapes, in most of the cases brings people to choose remote, cold, or arid areas consequently holding few species to protect [15].

This paper makes a contribution towards a better understanding of the linkage between PA, biodiversity conservation, ES, and human wellbeing, as a necessary step towards ensuring more effective and sustainable integration of ecological portion into other so many national development imperatives. It unpacks some of the home-grown realities of these linkages through the application of a multidimensional conservation approach. By doing so, it responds to five current weaknesses that are common in today’s conservation analysis in Rwanda as it has been raised by various authors [16–19]. These weaknesses are the following:

1. Focusing on the protection of certain preferred species instead of ecosystems. Yet PA are not meant to protect isolated species but the entire ecosystems.
2. Limited recourse to modern technology to correct the status quo that was established during the past when there was lack of adequate tools, even when the lack of objectivity and inefficiency are palpable.
3. Allowing much more attention to the direct income-generation side of conservation and leave behind the mission to transfer the natural heritage to the next generation in unimpaired state.
4. Sticking to the classic focus of PA on biodiversity failing to open the doors to the underway major shift toward broadening the goals of PA to also encompass the provision of ES for human well-being.
5. Disregarding the contribution of PA at addressing the planet’s concerning issues such as climate change, air pollution, disasters, epidemics, etc.

Due to the diffusion of remote sensing practices, ES mapping techniques have generated great interest in recent literature. Mapping provides spatial information about the state of ES which makes it an important element in the decision-making process where it is necessary to implement and monitor environmental policies [20, 21]. We hypothesize that the application of these newly developed methods on a region in which the majority of PA were created nearly a 100 years ago could highlight loopholes that lie between the established PA and both priority areas for conservation and new conservation imperatives, hence contribute to the establishment of a more efficient system. To test this hypothesis, we intend to combine both the mapping of endangered wildlife and of some key ES, quantify their level of overlapping with the existing NP system, to derive its efficiency against current imperatives, only after that can we be able to pinpoint the location of the main loopholes that exist through the system, to enlighten those who have the will and power to improve the conservation system in Rwanda.

## 2 Material and methods

### 2.1 Study area: Overview of rwanda and its NP system

**Fig 1:**
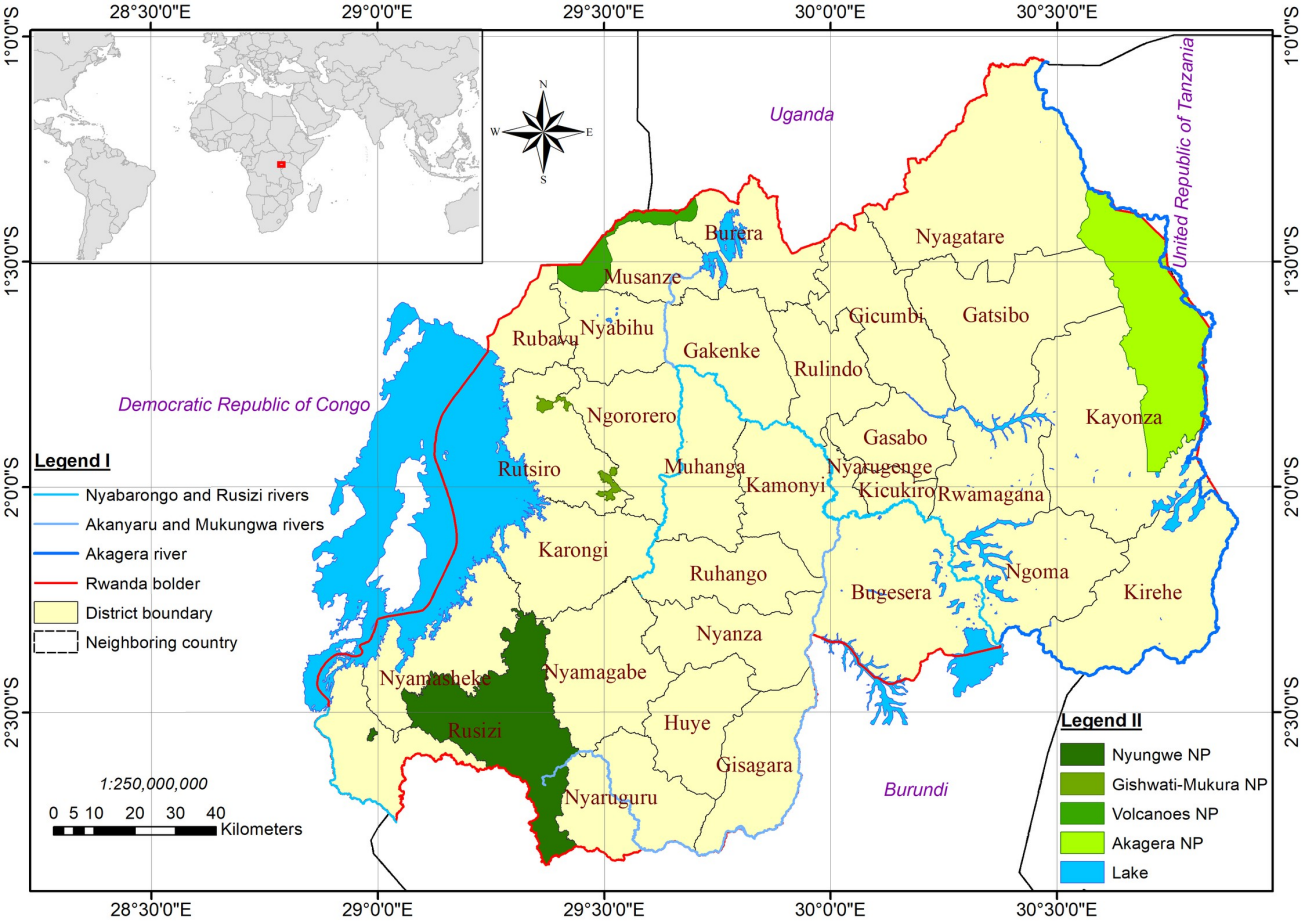
The map depicting the study area and its NP system.

Rwanda (Fig 1) is a landlocked country in central eastern of Africa at latitude 1°57′12.77″S and longitude 30°05′32.52″ E (Capital Kigali), its total land area is 26,338 sq.km, of which around 8.5% is covered by PA [22].

The country had a projected human population of 12,089,720 in 2018 and the highest population density on mainland Africa (477 people per sq.km) [16]. Around 90% of its still rapidly growing population depend on small-scale agriculture which exacerbated the quest for arable land and fuelwoods, leading to massive degradation of all forests and wetlands outside reserves [19]. Though located within the equatorial climatic zone, Rwanda is characterized by a tropical temperate climate due to its high altitude ranging from 950 m above sea level (a.s.l) at the Rusizi River (south-west) to 4507 m a.s.l at Mount Karisimbi (north-west). The area exhibits two dry seasons from January to February and from June to August, and two rainy seasons from March to May and From September to December. The annual rainfall increases in general from east to the west and ranges from less than 1000 mm per year to over 1800 mm per year. The temperature regime is more or less constant with a mean temperature of 20–24°C for the eastern lowlands, 18–21°C for the central plateau region and 16–17°C for the western high altitudes [23]. Diverse Eco-climatic zones comprising the Afro-alpine, humid, sub-humid and the sub-arid zones influence a large variety of vegetation and landscapes that support habitats for a wide range of animals some of which are endemic. Several bird species many of which have adapted to the human-modified habitats have been identified, many mammal, reptile amphibian and invertebrate species exist in large numbers as well.

Rwanda’s contemporary biodiversity conservation strategy has principally centered on PA model. The PA in Rwanda are largely a colonization legacy predominantly sustained by government institutions and international NGOs. One of the drawbacks of that PA model is that it can indirectly confine the arena for conservation action within reserves, hence condemning outside landscapes to maximum exploitation, which finally creates pockets of PA in extensive human transformed habitats [16]. In developing countries, PA model faces another more fundamental problem which is that the designation status, even when backed by laws, does not guarantee long term conservation, as it may be undermined by socio-political and economic changes and end up through downgrading, downsizing, de-gazettement or total degeneration [3]. Since their establishment, most of the Rwandan PA have lost at least half of their size principally due to farming and human settlement.

### 2.2 Rwanda PA establishment and management

We compiled the best available data on PA in Rwanda and their official boundaries. By the end of 2019, there was a total of 5 PA in Rwanda, including 4NP: Akagera National Park (ANP), Volcanoes National Park (VNP), Nyungwe National Forest (NNP), and Gishwati-Mukura National Park (GMNP). The first establishment of PA in Rwanda was during the colonial period in 1923, starting with the mountain forests: Mukura, Nyungwe, and Gishwati, and closely followed by the gazettement of the VNP in 1925 and the ANP in 1934 [22]. NNP was established by the law N° 22/2005 of 21/11/2005 and gazetted on 15 January 2006 [24]. The new boundaries of ANP were established by the law N° 33/2010 of 24/09/2010 and gazetted on 14 October 2010 [25]. The GMNP was established by the Law N°45/2015 of 15/10/2015 and gazetted on 01 February 2016 [26, 27]. Besides the NP, Rwanda possesses Rugezi Marsh that was declared a Ramsar Wetland of International Importance on 1^st^ December 2005. Some 82 bird’s species have been recorded within the marsh and its vicinity [28]. Before colonization, the conservation practice in Rwanda was no different to other many parts of Africa, firmly rooted in a body of beliefs, such as the totemic system that had extended the perception of the self to animal and plant species [29]. Later after the country’s independence in 1962, PA management duty was attributed to the Rwandan Office of Tourism and National Parks (ORTPN) in 1973, the institutional reformation in 2013 revised ORTPN’s old assignment into the new mandate of the department of tourism and conservation of the Rwanda Development Board (RDB) [30].

### 2.3 Rwanda ‘s priority habitats, biodiversity hubs and mapping

#### 2.3.1. Identification of Rwanda’s priority habitats & mapping

To establish Rwanda’s priority habitats types, we considered six established categories as important Rwandan habitat types to protect. They are: Urban areas, High mountain tops, Non-protected natural forest, Lakes and river + buffer zone, Swamps + buffer zone, PA. For each of these habitats we have assigned weights values according to their priority and ecological importance. Sites were ranked on the basis of three simple prioritisation protocols: 1) Species richness; 2) An index derived from the predominance of species with IUCN Red List status {IUCN Red List Categories are: EX—Extinct, EW—Extinct in the Wild, CR—Critically Endangered [CR(PE)—Critically Endangered (Possibly Extinct), CR(PEW)—Critically Endangered (Possibly Extinct in the Wild)], EN—Endangered, VU—Vulnerable, LR/cd—Lower Risk/conservation dependent, NT—Near Threatened (includes LR/nt—Lower Risk/near threatened), DD—Data Deficient, LC—Least Concern (includes LR/lc—Lower Risk, least concern)} [31]; 3) An irreplaceability index used for determining the priority of sites for conservation [32]. The scores -1, 1, 2, 3, 4, and 5 were assigned to describe the category’s relative importance. Urban area was assigned with a negative weight (penalization), because it is considered as the source of pollution that might cause future degradation of habitat quality.

Forests play a vital role for biodiversity conservation, and provide incentives to solve societal and economic well-being, whether through offering food and wood, creating income and employment, or by providing environmental services such as water and soil conservation, and climate mitigation [33]. Particularly, tropical forests significance in the global carbon cycle has led to the consideration and recognition of forest-based climate change mitigation measures in the international climate negotiations, agreements and policy frameworks [34] such as Reduced Emissions from Deforestation and Degradation (REDD) [35]. Tropical wetlands are also one of the most carbon (C) rich ecosystems in the world, their organic-rich soils contain exceptional large C stocks, damage to these fragile ecosystems, which rather take thousands of years to build up becomes a major source of greenhouse gas emissions. Besides, the loss of aboveground biomass and decomposition of organic material following their green cover’s disturbance causes the release of considerable amounts of CO_2_ to the atmosphere. Their vulnerability to land use, and numerous other ecosystem services (ES) they provide make wetlands a point of increasing interest for participation in climate change mitigation strategies, such as Reduced Emissions from Deforestation and Degradation (REDD+) [36, 37].

#### 2.3.2. Identification of Rwanda biodiversity hubs & mapping

We identified Rwanda’s biodiversity concentration stages at district level. This assignment wouldn’t be settled without recourse to the official shapefile of the administrative division of Rwanda into 30 districts. We have compiled an exhaustive biodiversity inventory data from a range of sites in different districts of Rwanda, and subdivided living organisms into 6 taxonomic groups: Mammals, Birds, Fish, Reptiles, Amphibians, and Plants. We unfortunately could not include invertebrates, even though they represent at least 95% of all species in East African region due to the fact that they are still largely undocumented [38]. To ensure the comprehensiveness of our catalogue, we have reviewed published and unpublished literature respective to plant and animals in different ecosystems of Rwanda. To map habitat distribution of threatened species in Rwanda, we have recorded the district in which the species were spotted or the location mentioned in the species description by different literature. For mammals, Sun, Bariyanga, and Wronski [39] provided an avant-garde document, the overview of available literature into historical context on mammals’ conservation in Rwanda. Amongst the most evocative ones, we have consulted Mammals of Africa, one of the most comprehensive mammalian inventories that have been conducted on the African continent. By documenting more than 10,000 species of mammals with illustrations, the book highlights the richest diversity of mammals on the continent [40]. We have also consulted the first mammal catalogue for Rwanda [41] and the first scientific exploration reports on the mammalian community in PA. [42–45]. We have also consulted the most recent report with a clear focus on large mammalian species [17, 46–49]. Beyond Mammals, other taxonomic groups were covered by ‘The bird & Mammal lists of Nyungwe National Park’ [50], The biodiversity of Buhanga relict forest [51], Inventory and mapping of threatened remnant terrestrial ecosystems outside PA through Rwanda [52], Biodiversity inventory for key wetlands in Rwanda [53]. The fifth national report to the convention on Biological diversity [54], and the study to establish a national list of threatened terrestrial ecosystems and species in need of protection in Rwanda [55]. The final selected list contains a total number of 810 species, including 217 plants, 142 mammals, 368 birds, 16 fish, 35 amphibians, and 32 reptiles. For the details of all species evaluated, see the S1 Appendix.

### 2.4 Rwanda’s threatened species list and mapping

#### 2.4.1. Rwanda’s list of threatened species

The level of threat against particular species was also highly considered. We couldn’t think of any better indicator of the health and status of species than the world’s most comprehensive information source on the global extinction risk status of animal, fungus and plant species namely the International Union for Conservation of Nature’s Red List of Threatened Species (IUCN Red List) for Rwanda, under different categories such as Extinct (EX), Critically Endangered (CR), Endangered (EN), and Vulnerable (VU). IUCN revealed that 26% of mammals, 14% of birds, and 41% amphibians are threatened globally [56]. In order to further enrich and complement our list, we have judged it more beneficial to add more information about the species survival threat caused by international trade in specimens of wild animals and plants as indicated by the Convention on International Trade in Endangered Species of Wild Fauna and Flora (CITES) [57].

#### 2.4.2. Mapping Rwanda’s threatened species

The endangered species from all taxonomic groups are supposedly a portion of a long list of species hence, we have assumed that the thematic maps of Rwanda’s threatened species represent a sub-set of the thematic maps of a given taxonomic group. No threatened species will appear in different locations than the locations of the species themselves.

### 2.5 Ecosystems service mapping

Conservation efforts have difficulty obtaining financial and social support for PA that do not demonstrate their tangible benefits for society [58]. ES are the direct and indirect contributions of ecosystems to human well-being. They directly or indirectly support human survival and quality of life [59]. The Millennium Ecosystem Assessment (MA), a major UN-sponsored effort to analyze the impact of human actions on ecosystems and human well-being, identified four major categories of ES: provisioning, regulating, cultural and supporting services. An often-heard challenge is that not all ES can be mapped, the most frequently mapped are provisioning ES (food, timber, water, etc.) and regulating ES (climate regulation) but cultural ES are less so [60]. As for supporting services, they are necessary ES for the production of all other ES. Some examples include biomass production, production of atmospheric oxygen, soil formation and retention, nutrient cycling, water cycling, and provisioning of habitat [61].

In developing world countries where data gaps are a major challenge the set of ES possible to analyze is generally constrained by the availability and consistence of spatial data at national scale, the cost, processing time, and level of expertise. Tools used in our investigation include Co$ting Nature (CN), Water World (WW) and ESRI’s ArcGIS 10.2.2. For a complete review of ES assessment tool, please see [62–65], forCN and WW, and [66] for ArcGIS 10.2.2. WW/CN are particularly attractive with the simplicity of the tools and wealth of ES related data available with them. Among their strengths over other site-scale ES tools is that they are web-based, freely available to users, does not require special GIS expertise, complete analysis in less time, have 1ha to 1 km^2^ spatial resolution, have monthly/diurnal temporal resolution, supply required biophysical data, provide data for climate change impacts, land use change impacts, land management impacts, water management impacts scenarios, and provide results about baseline services such as water quality, water quantity, water regulation, carbon, etc.

In this study, we assessed four key regulating ES: Carbon storage, Water balance, Human footprint on water quality/diarrheal disease and Human footprint on water quality/annual. These services were selected based on their significance to Rwandan context and the availability of data. All spatial analysis was conducted and maps generated respectively using ESRI’s ArcGIS 10.2.2 [66], CN and WW.

#### 2.5.1 Carbon storage

Carbon storage refers to the amount of carbon seized by terrestrial ecosystems (both aboveground and belowground) [9], the major carbon reservoirs are estimated to be oceans (38000 Pg C), terrestrial vegetation (500 Pg C), soils (1500–2000 Pg C), fossil fuels (5000–10000 Pg C) and atmosphere (780 Pg C); the exchanges of carbon between the atmosphere and terrestrial ecosystems is critical to the patterns of CO_2_ concentration in the atmosphere [67]. The natural stock of carbon benefits humans by reducing or stabilizing the atmospheric greenhouse gas concentration and mitigate climate change [58]. By considering different land use/land cover the carbon storage in Rwanda’s natural forest, non-forested, wetland and water bodies the carbon storage was estimated and its map was drawn.

We downloaded 4 satellite images taken by Landsat 8 OLI/TIRS from the U.S. Geological Survey Earth Explorer, tool that gives users the ability to query, search, and order aerial photographs, satellite images and cartographic products. The images were subtracted from the following address: Path 172 Row 61; Path 172 Row 62; Path 173 Row 61; Path 173 Row 62 and the date range was from 01/01/2019 up to 31/12/2019. Images with less than 10% of cloud all captured during the same dry season were preferred to minimize natural landscape elements fluctuations related to seasonal change. The most recent Landsat 8 mission carries the Operational Land Image (OLI) and the Thermal Infrared Sensor (TIRS). OLI/TIRS data are delivered at 30 m resolution with 11 spectral bands. The images are georeferenced and hold many layers containing data collected along both visible and invisible light spectrum that can be manipulated to reveal hidden indications about pictured ground features as highlighted in Table 1. The richness of Landsat archive combined with a no cost data policy, gave users an unequalled opportunity to monitor the natural and human-induced changes on the global landscape [68].

**Table 1:** Description of uses of Landsat bands (based on [68]).

Band name	L8 OLI/TIRS	Bandwidth (μm)	Resolution (m)	Description of use
Coastal/ Aerosol	Band 1	0.43–0.45	30	Coastal areas and shallow water observations; aerosol, dust, smoke detection studies.
Blue (B)	Band 2	0.45–0.51	30	Bathymetric mapping; soil/vegetation discrimination, forest type mapping, and identifying manmade features.
Green (G)	Band 3	0.53–0.59	30	Peak vegetation; plant vigor assessments.
Red (R)	Band 4	0.64–0.67	30	Vegetation type identification; soils and urban features.
Near-Infrared (NIR)	Band 5	0.85–0.88	30	Vegetation detection and analysis; shoreline mapping and biomass content.
Shortwave Infrared-1 (SWIR-1)	Band 6	1.57–1.65	30	Vegetation moisture content/drought analysis; burned and fire-affected areas; detection of active fires.
Shortwave Infrared-2 (SWIR-2)	Band 7	2.11–2.29	30	Additional detection of active fires (especially at night); plant moisture/drought analysis.
Panchromatic (PAN)	Band 8	0.50–0.68	15	Sharpening multispectral imagery to higher resolution.
Cirrus	Band 9	1.36–1.38	30	Cirrus cloud detection.
Thermal (T)	Band 10	10.60–11.19	100	Ground temperature mapping and soil moisture estimations.
	Band 11	11.50–12.51	100	

We have performed radiometric and geometric calibration on downloaded images to correct and eliminate distortions caused by the Earth’s rotation and camera angles. We have combined the multiple raster datasets into a single Mosaic image on which we have performed the supervised land use/cover classification, the common technic of remote sensing in which the image processing software (ArcGIS) is given primary guidance by the operator to specify the land use/cover classes of interest. We established the distribution of primary land use/cover classes following [34]. The familiarity with remote sensing enables us to explore beyond features that are recognizable when illuminated by natural light using appropriate band combination adjustments. Based on Horning [69], we have applied of some common band combinations applied to Landsat 8 images displayed as a red, green, blue (RGB) to detect and distinguish different land use/land cover:

Red = 4, Green = 3, Blue = 2: Natural Color
Red = 7, Green = 6, Blue = 4: False Color (urban)
Red = 5, Green = 4, Blue = 3: Color Infrared (vegetation)

Red = 6, Green = 5, Blue = 4: Vegetation Analysis
Red = 5, Green = 6, Blue = 2: Healthy Vegetation
Red = 5, Green = 6, Blue = 4: Land/Water

#### 2.5.2 Water balance

Water balance refers to water resources available in a catchment for different purposes including, but not limited to, agriculture and domestic purposes. The flow results coupled with the basin characteristics (slopes and imperviousness) can also be used in planning for watershed management measures including but not limited to erosion control, soil moisture and land management measures [23]. The accurate measurement of water balance is a rigorous exercise, requiring extensive site-based survey and measurements river water flow and other components is thus time consuming and expensive. Various approaches for measuring water balance exist, typically having to sacrifice accuracy for the sake of affordability. Rapid, spatially-explicit ES modeling tools such as Co$ting Nature or WaterWorld show promise for providing information about ES across multiple sites, to support decision making. Until such time comes where all the prerequisites are gathered desktop analyses relying on third party data remains the only method available.

#### 2.5.3 Human footprint on water quality/diarrheal disease and Human footprint on water quality/annual

Conservation initiatives rely on spatially explicit data on the location and distribution of ES to ensure that co-benefits are fully provided by nature to the people, hence the need to map them to help guide the most effective conservation investments [64]. We used WW, a fully distributed, process-based hydrological model, that utilizes remotely sensed and globally available datasets to support hydrological investigation and decision-making globally especially in data-poor environments. We’ve run the WW’s web browser-based interface (www.policysupport.org/waterword) at 1 sq.km resolution. All data required for model application in Rwanda as well as anywhere in the world are provided unless substituted by user’s data [70]. It is assumed that if a model is capable of reproducing current conditions based only on physical relationships, it is likely to continue to do so under scenario conditions where the physical relationships remain the same [71]. WW uses the metric human footprint on water quality as a proxy for water contamination [70]. Human footprint on water quality calculates the potential water quality in a grid cell, for each grid cell the human footprint index represents the percentage of total rainfall water coming from upstream that is influenced by sources of contamination. This means that the areas with extensive human activities such as agriculture or urban areas will leave a significant footprint on water downstream but can also be diluted by waters from PA or undisturbed areas.

## 3 Results

### 3.1 Biodiversity distribution by taxonomic group

#### 3.1.1 Distribution of biodiversity-rich habitat

**Fig 2:**
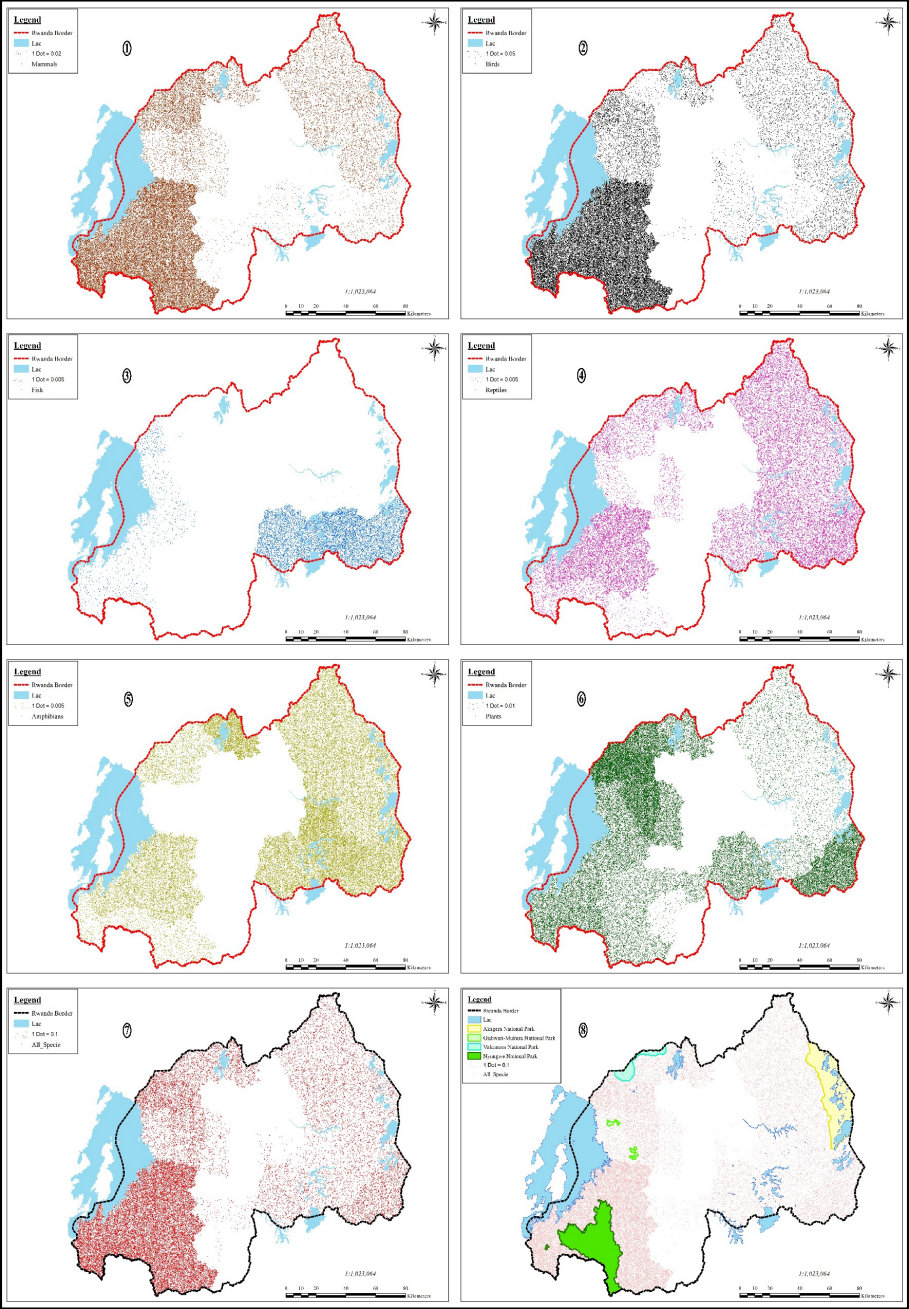
The biodiversity geographic distribution and richness by taxonomic group: ①Mammals, ②Birds, ③Fish, ④Reptiles, ⑤Amphibians, ⑥Plants, ⑦All species, ⑧PA.

Important biodiversity hubs for different taxonomic groups are shown in (Fig 2). The diversity of plants, amphibians, reptiles, mammals, and birds disproportionately span on most of the Rwandan territory, but various fish species are concentrated mainly in the southern lakes of Rwanda and in the lake Kivu and its numerous islands’ shores. An extreme poverty of biodiversity, in all taxonomic groups is very obvious in the central part of the country, the very region that holds the largest urban centers including the capital city Kigali, and Muhanga, Nyanza, Ruhango, Nyamata, and Rwamagana towns. The region is also characterized by intense anthropogenic activities and very rare remnants of natural habitat.

Important biodiversity hubs are distributed from east to the west of Rwanda, strongly subjected to the altitude and general annual rainfall distribution, hence by the 4 local climatic regions of Rwanda. From the east to the west, the lower elevation averaged 1220 m in the swampy Akagera river valley lays the ‘East-Rwandan dry and hot lowland zone’ in which the annual rainfall ≤900 mm and the annual temperature averaged at 21°C, is dominated by savannah habitats and is mainly conquered by large mammals most of which are protected in the ANP and others scattered through the surrounding districts in relatively small unprotected natural habitats such as Ibanda-Makera natural forest (Kirehe district), Karama natural forest (Bugesera district) and Mashoza natural forest (Ngoma district). Follows the interior highlands that consist of rolling hills and valleys over which the annual rainfall reaches 1200 mm is distinctly higher than in the eastern territories and the annual temperature averaged at 19°C which makes local climatic conditions become the ‘Temperate zone of the central highlands’ [72]. The highest lands in the west culminates at Karisimbi (4507 m) one of the 5 volcanoes of Birunga mountains on the extreme north of the Congo-Nile divide, the chain of mountains of rugged beauty that runs on the north-south axis and forms part of the Congo-Nile divide. This region receives the highest annual rainfall fulfilling the conditions of ‘Rwandan humid mountain climate’ with rainfall >1600mm and the annual temperature averaged at 16°C, which makes it home to the highest diversity of tropical plants, hence offering habitat to the most diverse fauna species in almost all taxonomic groups. This region holds the highest number of PA such as Nyungwe NP, Gishwati-Mukura NP, and Virunga NP but also the unprotected natural forests like Busaga natural forest (Muhanga district), and Karehe-Gatuntu forest complex (Karongi district). On the farthest west of Rwanda, the elevation drops to a low-lying depression west of the Congo-Nile divide along the shores of Lake Kivu to create a distinct regional climate ‘Kivu lake climate’ characterized by rainfall of about 1200 mm and annual temperatures around 20°C. The extreme south of this region is Bugarama plain, Rwanda’s lowest point at 900 m.a.s.l, which is part of the tectonic depression of the African Rift Valley. This region is home to more or less the same plant and animal species than in the ‘Temperate zone of the central highlands’.

#### 3.1.2 Priority areas for securing threatened species habitat

The geographical distribution of threatened species hotspots is linked to the geographical distribution of biodiversity-rich habitats, hence on the four peripheries of Rwanda at distances from the centroid of Rwanda that harbors the most important human activity centers of the country. Threatened species hotspots are mainly located in already existing PAs, that mainly extend on three peripheries of the country, with ANP on the extreme east, VNP and Rugezi wetland on the northern periphery and GMNP and NNP on the Western periphery of Rwanda. Moreover, part of threatened species is also located on the unprotected southern periphery of Rwanda. PAs have enormously contributed to save a large number of endangered species from extinction: ANP accommodates various endangered species such as the Critically Endangered (CE) *Osyris lanceolata (African sandalwood)*, the CE *Balaeniceps rex (Shoebill stalk)*, the CE *Giraffa camelopardalis (Giraffe)*, the Endangered (E) *Albizia amara (Bitter albizia)*, etc. the VNP offers habitat to the famous *CE Gorilla beringei beringei (Mountain Gorilla)*, and protection to the Vulnerable (VU) *Vaccinium stanleyi*, etc. NNP is home to the CE *Xyris vallida*, the E *Tabernaemontana odoratissima*, the E *Casearia runsorica*, the VU *Kupeornis rufocinctus (Red-collared Mountain Babbler)*, the E *Cercopithecus ascanius (Redtail monkey)*, etc. GMNP protects the E *Cercopithecus mitis kandti (Golden Monkey)*, The VU *Cryptospiza shelleyi (Shelley’s Crimson-wing)*, the E *Casearia runsorica*, etc. Rugezi wetland in Burera district harbors many endangered bird species such as the VU *Calamonastides gracilirostris (Papyrus Yellow Warbler)*, the E *Bradypterus graueri (Grauer’s Swamp warbler)*, the E *Balearica regulorum (Grey Crowned Crane)* and a considerable number of endemic amphibians such as the VU *Hyperolius castaneus (Brown reed frog)*.

Neverthlesss, a portion of threatened species also exist or exclusively exist in non-protected less undisturbed zones so far, particularly on the country’s southern periphery. The waters and shores of lake Kivu (Rubavu, Rutsiro, Karongi, Nyamasheke and Rusizi districts) and its numerous islands harbor various thematic groups of biodiversity, though its fish biodiversity is low (only harbors 26 fish species of which 15 are endemic Haplochromine), its islands are particularly important for the survivorship of water birds species who use the islands for reproduction, feeding and resting and are home to many registered endangered species on IUCN red list such as the E Ardeola idea (Madagascar Pond Heron). Rweru-Mugesera wetland complex in Bugesera, Ngoma, and Rwamagana districts accommodates ecological highly important *Cyperus papyrus* marshes which harbors the Nearly threatened (NT) *Laniarius mufumbiri (Papyrus gonolek)*, the CE *Thalassornis leuconotus (White-backed Duck)*, the CE *Netta erythrophthalma (Southern Pochard)*, the CE *Microparra capensis (Lesser Jacana)*, the E *Serinus koliensis (Papyrus Canary)*, the E *Necrosyrtes monachus (Hooded vulture)*, the VU *Pitta angolensis (African Pitta)*, the VU *Numida meleagris (Helmeted Guineafowl)*, etc. Buhanga natural forest in Musanze district contains the CE *Terathopius Ecaudatus (Bateleur)*, the CE *Polemaetus bellicosus (Martial Eagle)*, etc. Shagasha natural forest in Rusizi district contains the VU species like *Cercopithecus l’hoesti (L’hoest’s monkey)* isolated from other groups found in NNP. Muvumba natural forest in Nyagatare District is mainly constituted by *Acacia kirkii* an endemic plant species to Rwanda and not occurring elsewhere in the region. The forest also shelters the E bird species *Balerica regulorum (Grey crowned crane)* and the Vulnerable species such as *Balaeniceps rex (Shoebill)*. Ibanda-Makera natural forest in Kirehe district is a gallery forest associated with woodland and savannah in the east and papyrus swamp in the south toward Akagera river. The forest shelters the *CE Blighia unijugate (Triangle tops)*, the CE *Netta erythrophthalma (Southern Pochard)*, the VU *Francolinus afer (Red-necked Spurfowl Francolin)*, and the VU *Numida meleagris (Helmeted Guineafowl)*.

### 3.2 Ecosystem service distribution

The important areas for carbon storage are distributed mainly in places with forests in south-west, west, and north-west of Rwanda, these are respectively NNP (Rusizi, Nyamasheke, Nyaruguru, Nyamagabe, Karongi districts), GMNP (Ngororero, Rutsiro, Nyabihu, Rubavu districts), and VNP (Rubavu, Nyabihu, Musanze, and Burera districts); shrubs in east of Rwanda mainly in ANP (Gatsibo, Kayonza, and Nyagatate districts), Gabiro, Gako and Nasho military domains (respectively in Gatsibo, Bugesera, and Kirehe districts); wetlands mainly in the north and south-east of Rwanda, these are Rugezi wetland (Burera district) and Rweru & Mugesera wetland complex (Bugesera, Ngoma, Rwamagana districts); and lakes, mainly Kivu (Rubavu, Rutsiro, Karongi, Nyamasheke, Rusizi districts), Burera and Ruhondo (Burera and Musanze districts), Muhazi (Gicumbi, Gasabo, Gatsibo, Rwamagana, and Kayonza districts), Rweru & Mugesera (Rwamagana, Ngoma, and Bugesera district) and Ihema (Kayonza district). The amount of carbon storage corresponds to the amount of avoided flow of carbon into the atmosphere hence weakens further exacerbating effects to global climate change.

Important areas that contribute the most to the Annual water balance in Rwanda are generally located to higher elevation and are directly related to the level of the area’s green land cover. Sources and water upstream points are attributed higher values compared to the downstream of flow, hence their points of use are not as valuable as their points of production. Spatial distribution map shows highest values in the western part of Rwanda (Congo-Nile divide) that corresponds to higher elevation, most dense vegetation, highest annual rainfall, and the source of many water flows towards the Nile river in the east and Congo river in the west.

Rwanda has the highest population density on African mainland and human settlement is predominantly characterized by scattered pattern that spread on the country’s hills and highlands. The largest urban centers are located in the country’s center. Human footprint on water quality-diarrheal diseases is directly linked to human settlement, the map shows that PA such as NNP, VNP, ANP, Rugezi wetland and Rweru & Mugesera complex offer the opportunities of creation of safe water sources and effective sanitation systems that can guarantee the protection from contamination to diarrheal diseases.

**Fig 3:**
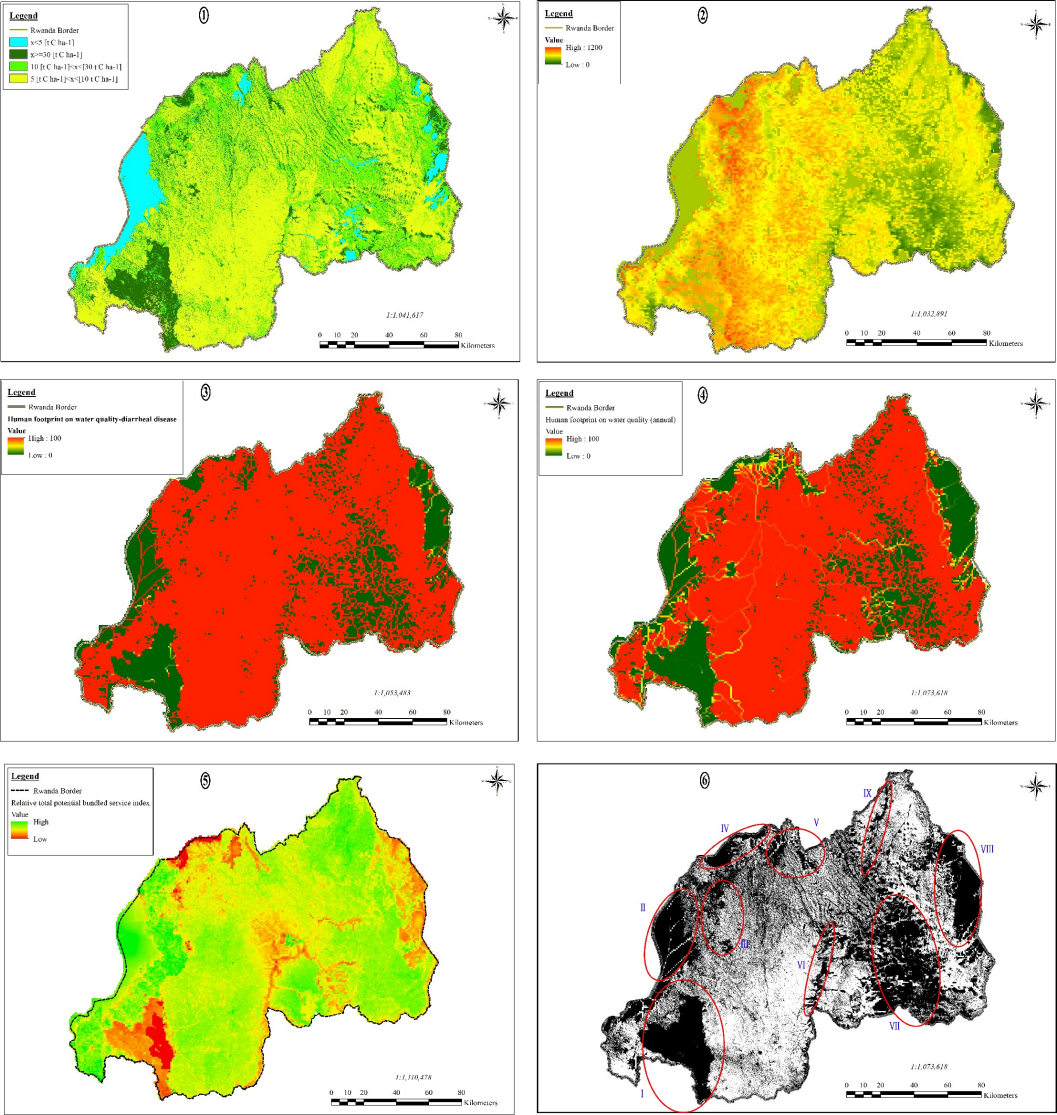
Distribution of ES: ①Carbon storage, ②Annual water balance, ③Human footprint on water quality-diarrheal diseases, ④Human footprint on water quality (annual), ⑤Relative total potential bundled service index, ⑥Priority areas for ES provision (I. NNP, II. Lake Kivu, III. GMNP, IV. VNP, V. Burera-Ruhondo Lake & Rugezi wetland, VI. Nyabarongo River wetlands, VII. Rweru-Mugesera wetland complex, VIII. Akagera NP, IX. Muvumba wetland).

Human footprint on water quality (annual) involves all sorts of pollutants that are linked to human activities that either worsen or degrade stream, river, lake, ocean, aquifer, or other body water’s turbidity or damage the water quality by rendering it toxic to humans or environment. Anthropogenic activities such as mining and quarries exploitation around wetlands and water bodies and industrial effluents from cities such as Kigali threaten by sedimentation and pollution the downstream water bodies due to poor sewage and inexistant waste treatment systems. On the other hand, the map (Fig 3) shows that PAs and other unprotected less undisturbed territories contribute less to human footprint on water quality (annual) but instead serve as retention pond that improve water quality before releasing it towards downstream water bodies.

## 4 Discussion

### 4.1 The delineation of NP system vs biodiversity hubs

While we don’t reject the fact that the biodiversity is generally well conserved and protected within PAs whilst out of them the biodiversity is highly threatened mainly due to intense human activities in Rwanda [54], this paper argues that the existing PAs system in Rwanda disproportionately privileges the protection of certain taxonomic groups and species over others, which leaves many species of some taxonomic groups out of the protection shield. Mammals and birds are respectively the most privileged and fish, reptiles and amphibians are respectively the least fortunate. Although the work of caring for the existing PAs is challenging and requires a lot of financial efforts, it is totally incomplete to think that nature preservation mission can achieve total success if it is only restricted with the sole conservation of biodiversity inside the PAs even for the most privileged taxonomic groups. Some unprotected key ecosystems are still scattered all over the country creating a reasonable porosity for biodiversity conservation in Rwanda [55]. Once they collapse, some species will go extinct and others very negatively affected especially those with limited dispersal mechanisms, small populations or restricted species. The creation of more reserves, setting up a conservation status to unprotected ecosystems to support the existing PA is of utmost importance to ensure the functionality and success of Rwanda’s protection network. A multilevel collaboration of stakeholders at national and international level, in which the public plays a strong role is crucial for the long-term conservation success in Rwanda. Beyond its heavily reliant on gorilla trekking and big five tourism, Rwanda needs to expand and diversify its tourism industry through the development of other forms of tourism, such as bird-related tourism and others [16]. The inclusion of unprotected habitats and other taxonomic groups into the protection scheme would undoubtedly set the foundation for Rwanda to better its environment, enrich its biodiversity and diversify its tourism. Well protected wetlands can play a particular role at enhancing the biodiversity of different taxonomical or functional groups, such as vegetation, amphibians, reptiles, fish, and birds. It is also recommended to enlarge that they can be ecologically viable.

### 4.2 The delineation of PA vs priority area for ES provision

Originally conceived to conserve iconic landscapes and wildlife, PAs are now expected to achieve an increasingly diverse set of conservation, social and economic objectives. Rwanda is a country with globally significant biodiversity and a high level of human dependence on ecosystems. We assessed key regulating ES (Carbon storage, Water balance, Human footprint on water quality/ diarrheal diseases and Human footprint on water quality/annual). PAs perform multiple roles that go beyond their intended conservation purposes, many of which go unnoticed and thus are seldom targeted for improvement. Such are the contribution to water balance, water quality, and carbon storage.

Mapping PA and ES do not only provide spatial information about their state to help targeting areas where it is necessary to implement and monitor environmental policies, but also facilitate understanding of the real impact of current environmental policies on ecosystems and the services they provide [21].

Rich biodiversity in protected wetlands can improve their treatment efficiency by enhancing their biogeochemical cycling [73] hence, enormously contributing to boost carbon sequestration hence, the reduction of carbon emissions. By contrast, urban areas are generally characterized by high concentration of nitrate, ammonia, and phosphate levels and are responsible for high concentrations of Ca, alkalinity, and T-hardness in the downstream rural area waters through erosions, surface and subsurface flow, and agricultural runoff. Higher nutrient levels and low concentrations of chemicals in the urban areas reveal a clear human footprint on water quality of their upstream river [74]. The widespread of human footprint on water quality shown is emphasized by the fact that Diarrhoea is a leading cause of childhood mortality in developing countries including Rwanda despite its simple protection measures, recent surveys indicate that 12% of children <5 years old nationally had caregiver-reported diarrhoea in the previous 2 weeks, 38% of children were stunted, and 2% of children were lost. Water quality is linked to diarrhoea through multiple pathways, the surveys also associate the time to get to water source and source of drinking water with the prevalence of childhood diarrhea in Rwanda [75, 76].

Given that mapping ES is becoming a key tool to guide decision making, the quality of such ES maps is important in order to be able to provide the most accurate information [77]. Our results corroborate the outcomes of the assessment that was conducted on China ecosystems which showed that improving ES can as well benefit the economic growth. Ouyang et al [78] provided more supporting cases from the United Kingdom, the United States of America, and Australia to reiterate that improvement of the provision of key ES can coexist with economic growth through intelligent policy design, although ES can decline without proper policies in place. Although the provision of ES has great value and benefits for local community and those living far away, it is unfortunate that these communities do not necessarily understand how much they benefits from them. The assessment of the contribution of ES to human wellbeing in western Rwanda reveals that the majority of services valued by forest-adjacent Rwandan inhabitants are not provided by tropical forests but by other habitats [19].

The method used for creating the maps for major groups of vertebrates and invertebrates has limitations as the method only identify the existence of the species in a given district but do not provide any information about specific species density, occupancy, threat and territorial range.

### 4.3 The delineation of PA vs biodiversity hubs & priority area for ES provision

Rwandan territory visibly contains numerous biodiversity hubs in which are concentrated exceptional level of endemic biodiversity. These zones are mainly but not limited to the officially established PAs (NNP, GMNP, VNP, ANP). On those can be added regions that had enjoyed some degree of protection against human intrusion during the last decades, but also remote & inaccessible areas (Lake Kivu, Burera-Ruhondo Lake & Rugezi wetland, Nyabarongo River wetlands, Rweru-Mugesera wetland complex, Muvumba wetland, etc.). Before enjoying a certain level of stability, it is important to mention that Landscape features (sinkholes, bedrock outcrops, water bodies, etc.), climatic conditions (temperatures, rainfall, etc.), and soil conditions (fertile soil, pH level, etc.) have in the first place combined to make them ‘ecological islands’ by giving them natural advantages allowing them to provide a convenient habitat, food, and nutrient to an exceptionally high number of biodiversity and provide high levels of ES. These precious regions are limited, fragile, and irreplaceable, anthropologic pressure and climate-driven may undermine the competitive advantage of these regions over others, hence the desperate need for protection. Rwanda’s NP system has been able to save many species that most probably wouldn’t survive until today. The expansion of the NP system to include the biodiversity hubs & priority area for ES provision would save the latter from further depletion and help the previous to achieve the PA’s modern time mission of nature conservation which does no longer only includes responsibilities on biodiversity protection but also upon ES.

## 5 Conclusion

Achieving the goal of protecting the optimal fractions of biodiversity in the high densely populated Rwanda, desperately requires tools that enable the existing NP system to support the most species at the least cost. In addition, the NP system needs to reform and embrace new modern conservation responsibilities that have significantly increased compared to those they were required to deliver to at their establishment a century ago. Including biodiversity hubs and priority areas for ES provision to the wider national protection system through green corridors and PAs widening would not only save them from further impairments, but also reduce the risk of extinction of many threatened species, and boost their provision of ES. The procedures shown in this study are the freely available based models for application in data-poor but problem-rich environments therefore, they are easily transferable to other regions for assessing the benefits of PAs beyond the conservation of biodiversity. Such assessments will allow the identification of areas that require further conservation actions, as well as those that are contributing to biodiversity conservation and ES provision. As such, our results directly respond to recent calls for efficient prioritization of the location of PAs. [6, 7].

## Supporting information

S1 Appendix (XLS)
